# Dipeptide YA is Responsible for the Positive Effect of Oyster Hydrolysates on Alcohol Metabolism in Single Ethanol Binge Rodent Models

**DOI:** 10.3390/md18100512

**Published:** 2020-10-11

**Authors:** Adrian S. Siregar, Marie Merci Nyiramana, Eun-Jin Kim, Eui-Jung Shin, Min Seok Woo, Jin-Mok Kim, Jung Hwan Kim, Dong Kun Lee, Jong Ryeal Hahm, Hyun Joon Kim, Chang-Woon Kim, Nam-Gil Kim, Si-Hyang Park, Yeung Joon Choi, Sang Soo Kang, Seong-Geun Hong, Jaehee Han, Dawon Kang

**Affiliations:** 1 Department of Physiology and Institute of Health Sciences, College of Medicine, Gyeongsang National University, Jinju 52727, Korea; adriansiregar46@gmail.com (A.S.S.); mariemerci1994@naver.com (M.M.N.); eunjin1981@hanmail.net (E.-J.K.); eui-jung@naver.com (E.-J.S.); whitewms@naver.com (M.S.W.); dklee@gnu.ac.kr (D.K.L.); hong149@gnu.ac.kr (S.-G.H.); jheehan@gnu.ac.kr (J.H.); 2 Department of Convergence Medical Science, Gyeongsang National University, Jinju 52727, Korea; kimhj@gnu.ac.kr (H.J.K.); kangss@gnu.ac.kr (S.S.K.); 3 Department of Clinical Laboratory Science, Masan University, Changwon 2640, Korea; jmkim@masan.ac.kr; 4 Department of Premedicine, College of Medicine, Gyeongsang National University, Jinju 52727, Korea; righteous_flame@gnu.ac.kr; 5 Department of Internal Medicine, Hospital and Institute of Health Sciences, College of Medicine, Gyeongsang National University, Jinju 52727, Korea; jrhahm@gnu.ac.kr; 6 Department of Anatomy and Institute of Health Sciences, College of Medicine, Gyeongsang National University, Jinju 52727, Korea; 7 Department of Obstetrics and Gynecology, Samsung Changwon Hospital, Sungkyunkwan University School of Medicine, Changwon 51353, Korea; kcwoon@naver.com; 8 Department of Marine Biology and Aquaculture and Institute of Marine Industry, Gyeongsang National University, Tongyeong 53064, Korea; ngkim@gnu.ac.kr; 9 Sunmarin Biotech, Jinju Bioindustry Foundation, Jinju 52839, Korea; hyangi51@hanmail.net; 10 Ocean-Pep, Jinju Bioindustry Foundation, Jinju 52839, Korea; yjchoi@gnu.ac.kr

**Keywords:** alcohol, inflammation, liver injury, oxidative stress

## Abstract

Accumulative alcohol hangovers cause liver damage through oxidative and inflammatory stress. Numerous antioxidant and anti-inflammatory reagents have been developed to reduce alcohol hangovers, but these reagents are still insignificant and have limitations in that they can cause liver toxicity. Oyster hydrolysate (OH), another reagent that has antioxidant and anti-inflammatory activity, is a product extracted through an enzymatic hydrolysis process from oysters (*Crassostrea gigas*), which can be easily eaten in meals. This study was aimed at determining the effects of OH on alcohol metabolism, using a single high dose of ethanol (EtOH) administered to rodents, by monitoring alcohol metabolic enzymes, oxidative stress signals, and inflammatory mediators. The effect of tyrosine-alanine (YA) peptide, a main component of OH, on EtOH metabolism was also identified. In vitro experiments showed that OH pretreatment inhibited EtOH-induced cell death, oxidative stress, and inflammation in liver cells and macrophages. In vivo experiments showed that OH and YA pre-administration increased alcohol dehydrogenase, aldehyde dehydrogenase, and catalase activity in EtOH binge treatment. In addition, OH pre-administration alleviated CYP2E1 activity, ROS production, apoptotic signals, and inflammatory mediators in liver tissues. These results showed that OH and YA enhanced EtOH metabolism and had a protective effect against acute alcohol liver damage. Our findings offer new insights into a single high dose of EtOH drinking and suggest that OH and YA could be used as potential marine functional foods to prevent acute alcohol-induced liver damage.

## 1. Introduction

Alcohol has been used as a means of socializing and as a beverage to relieve stress since ancient times. As the economic growth of modern society leads to the formation of a culture of bingeing and frequent drinking, alcohol consumption has increased and many social problems such as hangovers, liver dysfunction, and alcoholism have occurred. Alcohol absorbed by the gastrointestinal tract is metabolized in the liver, which is the organ most sensitive to alcohol in the human body. The liver has excellent regenerative and compensatory abilities, but acute or chronic alcohol in excessive amounts damages liver cells and causes liver disease without allowing time to regenerate (Alcohol-related liver diseases, NHS, UK). Repetitive heavy drinking or hangovers lead to accumulation of biochemical, oxidative, and inflammatory changes over long periods, causing more serious problems in the body. Hangovers occur more when individuals consume more alcohol than usual [1].

Alcohol hangovers have potential symptoms such as fatigue, thirst, drowsiness, headache, dry mouth, nausea, weakness, vomiting, and cognitive impairment [2,3]. However, the severity and temporal type of hangover symptoms vary markedly from person to person. These differences are related to the amount of alcohol consumed and immunity to hangovers [4,5]. Alcohol is metabolized to acetaldehyde by alcohol dehydrogenase (ADH), catalase, and cytochrome P450E1 (CYP2E1). Acetaldehyde is then converted by aldehyde dehydrogenase (ALDH) into acetate [6]. The causes of these hangover symptoms are known to be toxicity of alcohol and alcohol metabolites, production of free radicals, changes in neurotransmitters and immune factors, and increased inflammatory mediators. These factors are involved in hangover pathology and liver damage [7,8]. Substances that can relieve hangovers, especially those that can promote ethanol (EtOH) metabolism, may contribute to reducing liver damage.

Natural products such as vegetables, fruits, and traditional herbs have been developed as potential treatments for alcohol hangovers based on the findings that they promote the elimination of alcohol and alcohol metabolites, inhibit inflammatory mediators, and stimulate the expression of antioxidant enzymes [9]. However, they improve some symptoms associated with alcohol hangovers such as fatigue, nausea/vomiting, and gastric pain, but not all [10]. In addition, these products can cause liver toxicity depending on the individual’s health condition [11]. More research is needed to develop clinically effective hangover treatments. Recently, marine bio-related research has been growing worldwide, recognizing the ocean as a biological resource. 

Oysters (*Crassostrea gigas*) are a nutritious seafood that are often found in meals. Oysters have high concentrations of protein, various amino acids and vitamins, and minerals, and a low concentration of fat [12,13]. Polysaccharides and phenolic compound 3,5-dihydroxy-4-methoxybenzyl alcohol have been isolated from the water and alcohol extracts of oyster, respectively [14,15]. Different ingredients can be extracted from oysters according to the extraction method. For use as a dietary supplement, it is necessary to keep the extracted biologically active ingredients constant. Recently, oysters have been processed by enzymatic hydrolysis to improve their nutritional properties. Oyster hydrolysate (OH) contains a large amount of free amino acids and low-molecular peptides, and has high physiological activity compared to oyster extract [16]. OH has antioxidant and anti-inflammatory activity [17,18]. Amino acids enhance the activity of alcohol-metabolizing enzymes and reduce acute alcohol hangovers [19,20]. The β-thymosin peptide derived from oyster shows anti-inflammatory activity, inhibiting nitric oxide (NO) and prostaglandin E2 (PGE2) production and pro-inflammatory cytokine expression in LPS-induced RAW264.7 cells [21]. Tyrosine-alanine (YA) peptide is the main component isolated from oyster by an enzymatic hydrolysis process and has antioxidant and anti-inflammatory effects [22]. Little is known regarding the effect of OH and YA on alcohol metabolism.

Our current study aims to investigate the effect of OH on EtOH metabolism, in a single high dose of EtOH administered to rodents, by monitoring alcohol-metabolizing enzymes, oxidative stress, and inflammation. We also investigated the effect of YA, an indicator material for quality control and effectiveness assessment of OH, on EtOH metabolism. 

## 2. Results

### 2.1. Protective Effect of Oyster Hydrolysate on Ethanol-Induced Cell Death in Liver Cells and RAW264.7 Cells

#### 2.1.1. Antioxidant and Anti-Inflammatory Activity of OH

**Figure 1:**
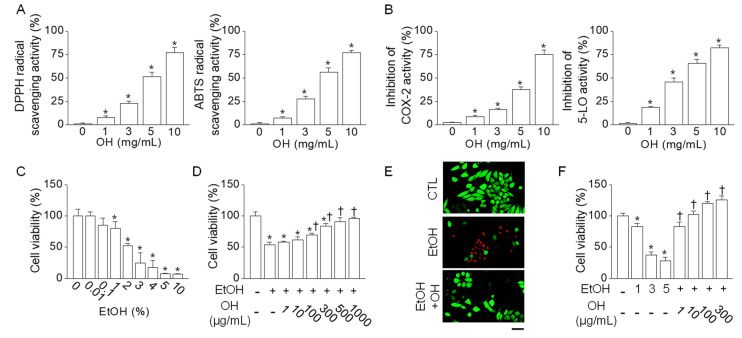
Antioxidant and anti-inflammatory activity of oyster hydrolysate (OH). (**A**) Antioxidant activity of OH. 2.2-diphenyl-1-picrylhydrazyl (DPPH) and 2,2-azinobis-(3-ethylbenzothiazoline-6-sulfonate) (ABTS) radical scavenging activity was measured. (**B**) Anti-inflammatory activity of OH. Cyclooxygenase-2 (COX-2) and 5-lipoxygenase (5-LO) activity was measured. OH activity was calculated by measuring absorbance. (**C**) Cytotoxic effect of ethanol (EtOH) on Chang liver cells. Cells were exposed to different concentrations of EtOH for 24 h. (**D**) Protective effect of OH on EtOH-induced cell death. Cells were pretreated with OH 1 h before EtOH treatment for 24 h. OH effect was examined in 2% (*v*/*v*) EtOH treated cells. (**E**) Live (carcein, in green) and dead (propidium iodide (PI), in red) cell staining in Chang liver cells. (**F**) Protective effect of OH on EtOH-induced cell death in a macrophage cell line RAW264.7 cells. Each bar represents mean ± SD of four independent experiments. * *p* < 0.05 compared to no treatment. ^†^ *p* < 0.05 compared to EtOH-only treatment. Scale bar represents 50 μm. The concentration of OH was expressed as the weight of lyophilized OH powder (*w*/*v*).

OH decreased 2.2-diphenyl-1-picrylhydrazyl (DPPH) and 2,2-azinobis-(3-ethylbenzothiazoline-6-sulfonate) (ABTS) chemical radicals. DPPH and ABTS radical scavenging activity was significantly increased dose-dependently by OH (n = 4, *p* < 0.05, Figure 1A). OH significantly inhibited the activity of cyclooxygenase-2 (COX-2) and 5-lipoxygenase (5-LO), biosynthetic enzymes related to inflammatory processes, in a dose-dependent manner (n = 4, *p* < 0.05, Figure 1B). 

#### 2.1.2. Inhibition of Ethanol-Induced Cytotoxicity by Oyster Hydrolysate

OH had no cytotoxic effect on the cell viability of Chang human liver cells. As shown in Figure 1C, EtOH induced cell death dose-dependently, and the cell viability with 2% (*v*/*v*) EtOH treatment was 53.5 ± 2.9%. Pretreatment with OH reduced EtOH-induced cell death in a dose-dependent manner (Figure 1D). EtOH and OH at the concentrations of 2% (*v*/*v*) and 100 μg/mL (w/v), respectively, were used for further experiments. Live/dead cell staining of Chang liver cells showed an increase in the number of propidium iodide (PI)-positive dead cells with the 2% (*v*/*v*) EtOH treatment. Cells pretreated with OH showed fewer dead cells than in the treatment with EtOH alone (Figure 1E). To further explain the effects of OH on the viability of immune cells and the mechanism of alcohol-induced inflammation, the effects of EtOH and OH were investigated in the macrophage cell line RAW264.7 cells. EtOH-induced cell death in RAW264.7 cells was recovered by pretreatment with OH in a dose-dependent manner (Figure 1F).

### 2.2. Antioxidant and Anti-Inflammatory Effects of Oyster Hydrolysate on Ethanol-Treated Chang Liver Cells

**Figure 2:**
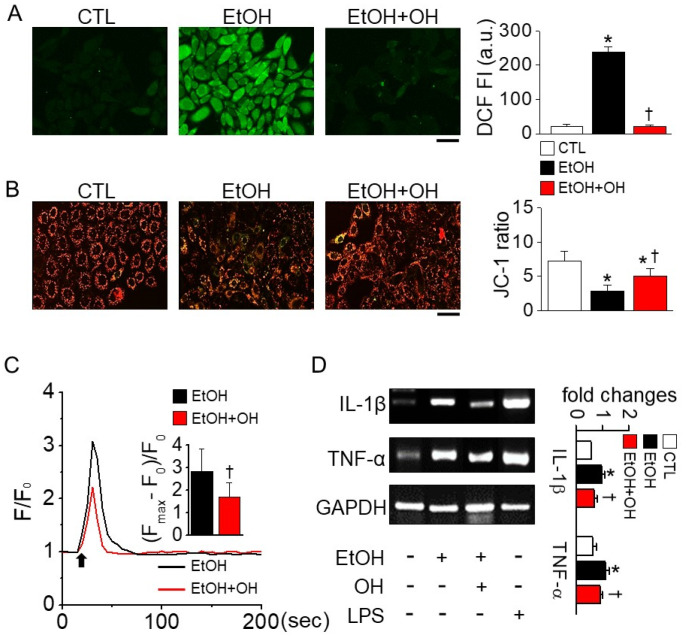
EtOH-induced oxidative stress reduced by OH. (**A**) Reduction of EtOH-induced reactive oxygen species (ROS) generation by OH. Chang cells were exposed to 2% EtOH for 5 h after pretreatment with OH for 1 h. (**B**) Protection of EtOH-induced changes in mitochondrial membrane potential (MMP) by OH. Cells were incubated with JC-1, a specific MMP dye, for 24 h. Cells were pretreated with OH before 1 h of EtOH treatment. Green and red indicate JC-1 monomers and JC-1 aggregates, respectively. (**C**) EtOH-induced increase in intracellular Ca^2+^ concentration reduced by OH pretreatment in Chang liver cells. Cells were loaded with fluo-3 AM for 45 min to evaluate changes in Ca^2+^ levels. EtOH (2%, *v*/*v*) was applied to the bath solution in the presence of CaCl_2_ (1 mM). Arrow represents addition of EtOH. (**D**) Reduction of EtOH-induced increase in interleukin 1 beta (IL-1β) and tumor necrosis factor alpha (TNF-α) mRNA expression by OH pretreatment. Each bar represents mean ± SD of four independent experiments. * *p* < 0.05 compared to control (CTL). ^†^ *p* < 0.05 compared to EtOH-only treatment. Scale bar represents 50 μm. F, fluorescence intensity. Calculation method is described in the Materials and Methods.

Chang liver cells were pretreated with OH (100 μg/mL) for 1 h before 2% EtOH treatment for 5 or 24 h. High amounts of reactive oxygen species (ROS) were detected in cells treated with EtOH for 5 h compared to control. The EtOH-induced ROS generation was significantly decreased in cells pretreated with 100 μg/mL OH by 90.9 ± 1.7% (n = 4, *p* < 0.05, Figure 2A). ROS generation was not detected in 24 h treatment with EtOH. 

Increased ROS generation can induce depolarization of mitochondrial membrane potential (MMP). Chang liver cells stained with JC-1 exhibited red and green fluorescence, and EtOH treatment shifted the color from red to green in the cells, whereas OH pretreatment returned the color to red (Figure 2B). OH pretreatment significantly reduced EtOH-induced MMP depolarization by 1.7-fold (n = 20, *p* < 0.05). Depolarization of MMP is related to an increase in intracellular Ca^2+^ concentration ([Ca^2+^]_i_). Pretreatment with OH significantly reduced EtOH-induced transient increase in [Ca^2+^]_i_ by 40% (n = 10, *p* < 0.05, Figure 2C). 

The expression of pro-inflammatory cytokines was observed to identify the anti-inflammatory effect of OH. EtOH treatment significantly increased interleukin 1 beta (IL-1β) and tumor necrosis factor (TNF-α) expression compared to control (n = 4, *p* < 0.05, Figure 2D). Lipopolysaccharide (LPS) treatment was used for induction of pro-inflammatory cytokines. OH pretreatment reduced the EtOH-induced increase in IL-1β and TNF-α expression levels by 28% and 18%, respectively (Figure 2D).

### 2.3. Effect of OH on EtOH Metabolism in a Single High Dose of Etoh Administered to Rats

#### 2.3.1. Effect of EtOH Binge Treatment on ALT and AST Liver Enzymes 

**Figure 3:**
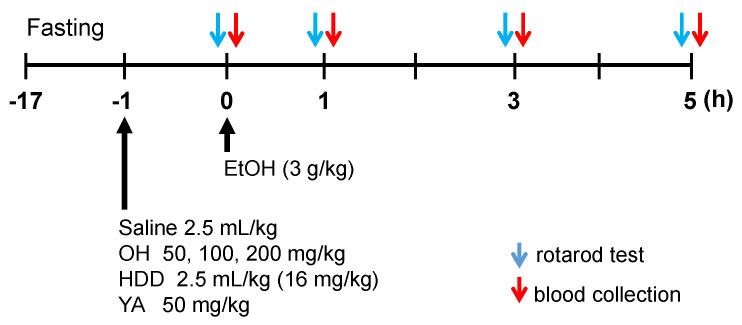
Animal experimental group for the studying alcohol intoxication. Mice and rats were administered a single high dose of EtOH on day 0. The actual dose for each treatment was calculated from the weights of the animals at the start of the experiments. Saline, OH, hovenia dulcis drink (HDD), and YA (tyrosine-alanine) were pre-administered 1 h before EtOH administration for vehicle (EtOH + saline), OH (EtOH + OH), HDD (EtOH + HDD), and YA (EtOH + YA) groups, respectively. All compounds were administered by oral gavage. Rotarod test was performed prior to blood collection at indicated times.

Treatment protocol for the experimental animal groups for the EtOH binge model is summarized in Figure 3. As shown in Table 1, among the three different concentrations (50 mg OH/kg BW (body weight), 100 mg OH/kg BW, and 200 mg OH/kg BW), the combination with a high concentration of OH (HOH, 200 mg/kg) significantly reduced alcohol concentration compared to EtOH-only treatment (n = 10, *p* < 0.05). However, there was no significant difference between EtOH + saline group and EtOH + HOH in the alanine aminotransferase (ALT) and aspartate aminotransferase (AST) levels. EtOH group showed a significant difference in the ALT level, but not in the AST level, compared to vehicle group (n = 10, *p* < 0.05). The high concentration of OH was used for further experimentation.

The experimental groups were further divided into four groups (n = 10 in each): vehicle, EtOH + saline, EtOH + OH (200 mg OH/kg BW), and EtOH + hovenia dulcis drink (HDD). HDD was used as a comparative substance because it has been studied widely as a hangover reliever [23]. Body and liver weights were measured at the beginning and end of the experiment, respectively. There were no significant changes in liver weight or AST level among the experimental groups (Table 2). In the ALT level, there was a significant difference between EtOH + saline group and EtOH + HDD group.

#### 2.3.2. Enhancement of Alcohol Metabolism by OH

**Figure 4:**
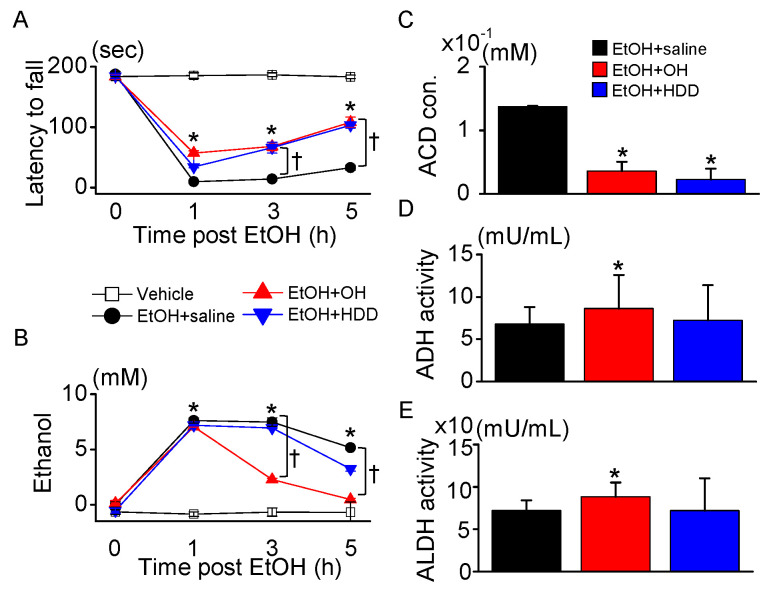
Effect of OH on hepatic enzyme activity. Concentrations of EtOH, its metabolite, and hepatic enzymes were measured in blood obtained from EtOH-intoxicated rats. (**A**) Rotarod test. Latency to fall was measured at three times after EtOH administration; 0 h indicates prior to EtOH administration. (**B**) Measuring blood EtOH concentration at 1, 3, and 5 h after EtOH administration. In A and B, each data point represents the mean ± SD of five independent experiments. * *p* < 0.05 compared to vehicle; ^†^ *p* < 0.05 compared to EtOH + saline. (**C**) Decrease in concentration of blood acetaldehyde (ACD) in OH and HDD pre-administered groups. (**D**,**E**) Activity of alcohol dehydrogenase (ADH) and aldehyde dehydrogenase (ALDH) measured at 5 h after EtOH administration. In C–E, each bar represents mean ± SD of five independent experiments. * *p* < 0.05 compared to EtOH + saline. Scale bar represents 50 μm.

Motor coordination and balance in rats administered EtOH were assessed by rotarod performance test at 0, 1, 3, and 5 h after EtOH administration. The EtOH group showed a significant decrease in the latency of rats to fall compared to the vehicle group, while the addition of OH (EtOH + OH group) improved the retention time of the rats on the rotating rod at all evaluation times compared to EtOH-alone (EtOH + saline group) (n = 10, *p* < 0.05, Figure 4A). Except for between 1 and 3 h after EtOH administration in the EtOH + saline group, the rotarod performance significantly increased over time in the three groups (EtOH + saline, EtOH + OH, and EtOH + HDD; *p* < 0.05).

EtOH concentrations were measured at 0, 1, 3, and 5 h after EtOH administration. The EtOH concentration in blood was high at 1 h after EtOH administration, then decreased over time (Figure 4B). The OH combination significantly decreased the EtOH concentration at 3 and 5 h after administration by 69.7 ± 2.0% and 90.9 ± 1.8%, respectively, compared to the EtOH + saline group, while HDD significantly decreased the EtOH concentration only at 5 h after administration by 37.8 ± 4.4% (n = 10, *p* < 0.05, Figure 4B). The reducing effect of OH on EtOH concentration appeared earlier and was greater than that of HDD. Except for between 1 and 3 h in the EtOH + saline and EtOH + HDD groups after EtOH administration, the EtOH concentration significantly decreased over time in the three groups (EtOH + saline, EtOH + OH, and EtOH + HDD; *p* < 0.05). 

OH was also effective in reducing blood acetaldehyde concentrations in EtOH-intoxicated rats (Figure 4C). The OH combination (EtOH + OH) significantly increased ADH and ALDH activity for EtOH and acetaldehyde metabolism compared to EtOH + saline group (n = 10, *p* < 0.05, Figure 4D,E). 

**Figure 5:**
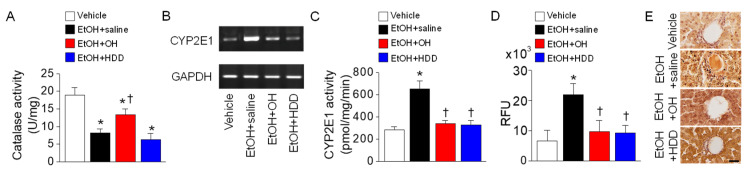
EtOH-induced up-regulation of CYP2E1 suppressed by OH. (**A**) Catalase activity. (**B**) CYP2E1 mRNA expression in liver tissues. (**C**) CYP2E1 activity in liver lysates. (**D**) ROS/RNS free radical activity. ROS generation was measured in liver tissue lysates obtained from rats exposed to 3 h EtOH. Free radical content in samples was determined by comparing with predetermined DCF standard curve. ROS and RNS species can react with dichlorodihydroflurescin (DCFH), which is rapidly oxidized to the highly fluorescent 2’, 7’-dichlorodihydrofluorescein (DCF). Fluorescent activity is expressed in relative fluorescence units (RFU). (**E**) Detection of oxidative stress in liver tissue. OxyIHC^TM^ detects protein oxidation. Scale bar represents 30 μm. Each bar represents mean ± SD of five independent experiments. * *p* < 0.05 compared to vehicle; ^†^ *p* < 0.05 compared to EtOH + saline group.

Catalase and CYP2E1 are involved in acetaldehyde metabolism. In the liver tissue isolated from the EtOH + saline group, catalase activity was decreased compared to vehicle group, whereas OH combination (EtOH + OH group) significantly decreased EtOH-induced reduction of catalase activity (n = 10, *p* < 0.05, Figure 5A). When CYP2E1 uses oxygen for alcohol metabolism, ROS are generated, causing oxidative stress and tissue damage [6]. In the liver tissue isolated from the EtOH + saline group, CYP2E1 mRNA expression level was highly increased, while OH and HDD combination groups showed lower CYP2E1 expression than the EtOH group (Figure 5B). Changes in CYP2E1 activity among experimental groups showed similar patterns to those in CYP2E1 mRNA expression. OH pre-administration significantly decreased CYP2E1 activity by 48.0 ± 1.7% (n = 10, *p* < 0.05; Figure 5C). The EtOH group showed a significant increase in ROS generation by approximately 4-fold, while the OH pre-administration group showed significantly reduced ROS generation by 56 ± 17% in liver tissue lysates (n = 10, *p* < 0.05; Figure 5D). Protein carbonyl content, a marker for oxidative stress, was also detected in liver tissues obtained from the EtOH group. OH pre-administration decreased the oxidative stress, as judged by reduction of black dots (n = 3, Figure 5E). 

**Figure 6:**
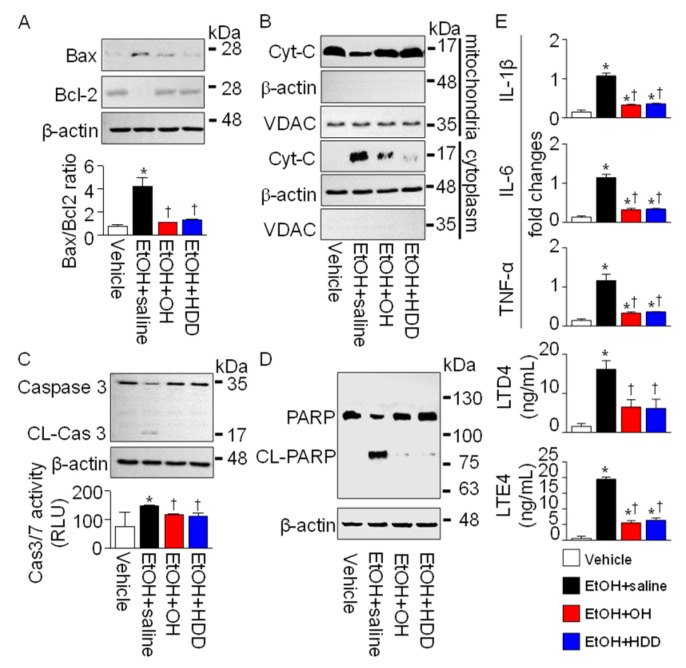
Effect of OH on apoptotic and inflammatory signals in liver tissue obtained from EtOH-intoxicated rats (5 h binge treatment). (**A**) Western blot analysis of proapoptotic Bax and anti-apoptotic Bcl-2. (**B**) Release of cytochrome C (cyto C) into the cytoplasm from the mitochondria in EtOH + saline group. Liver tissue lysates were fractionated into mitochondria and cytoplasm components. It is confirmed that voltage-dependent anion channel (VDAC) is expressed in the mitochondrial fraction, but not β-actin. Cell lysate (30 μg of protein per lane) was loaded on gel for immunoblotting. (**C**,**D**) Cleavage of caspase 3 and Poly (ADP-ribose) polymerase (PARP) in EtOH + saline group. Caspase 3/7 activity was expressed in relative light units (RLU). (**E**) Detection of inflammatory mediators. Changes in mRNA expression of IL-1β, IL-6, and TNF-α were detected in liver tissues using real-time PCR. Concentration of LTD4 and LTE4 was measured by immunoassay. Each bar represents mean ± SD of four independent experiments. * *p* < 0.05 compared to vehicle; ^†^ *p* < 0.05 compared to EtOH + saline group.

Oxidative stress-induced liver damage was identified by detecting apoptotic and inflammatory signals in liver tissues obtained from EtOH-intoxicated rats. EtOH-induced oxidative stress leads to hepatocyte death [24]. In the EtOH group, pro-apoptotic Bax protein increased, whereas anti-apoptotic Bcl-2 protein decreased. The Bax/Bcl-2 ratio increased significantly in the EtOH + saline group by 6.1 ± 1.9-fold, but the ratio was significantly decreased in the OH pre-administered group by 74 ± 4% (n = 3, *p* < 0.05; Figure 6A). The Bax/Bcl-2 ratio also decreased in HDD pre-administered group by 68 ± 4%. Mitochondrial release of cytochrome C into the cytoplasm was detected in the EtOH-administered group, and cytochrome C release was lower in the OH- and HDD-administered groups (n = 3, Figure 6B). Caspase 3 was cleaved in the EtOH-administered group, but the cleavage was prevented in the OH-pre-administered group. In addition, caspase 3/7 activity increased in the EtOH-administered group and was reduced in the OH- and HDD- pre-administered groups (n = 3, Figure 6C). Poly (ADP-ribose) polymerase (PARP) cleavage was detected in the EtOH-administered group, but PARP cleavage was attenuated in the OH and HDD pre-administered groups (n = 3, Figure 6D). Real-time PCR data showed a significant increase in expression of pro-inflammatory cytokines (IL-1β, IL-6, and TNF-α) in the EtOH-administered group compared to the vehicle group (n = 4, *p* < 0.05). The EtOH-induced increase in pro-inflammatory cytokines was significantly reduced in the OH and HDD pre-administered groups (n = 4, *p* < 0.05, Figure 6E). The primer sequences for real-time PCR are shown in Table 3. Leukotriene D4 (LTD4) and leukotriene E4 (LTE4) were increased in the EtOH-administered group and were significantly reduced in the OH and HDD pre-administered groups (n = 4, *p* < 0.05, Figure 6E).

#### 2.3.3. Contribution of YA dipeptide to the Enhancement of Alcohol Metabolism

**Figure 7:**
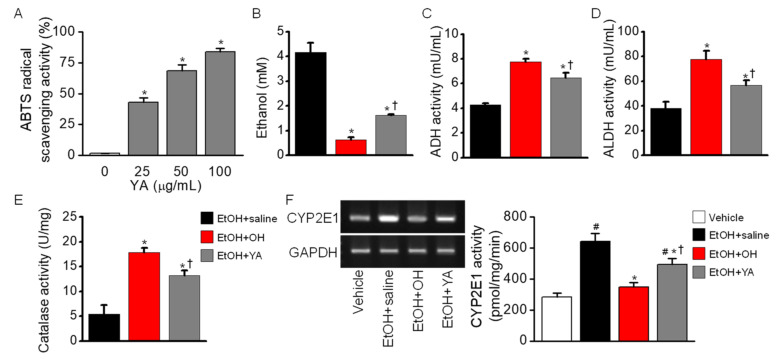
Effect of tyrosine-alanine (YA) dipeptide on EtOH metabolism. (**A**) Dose-dependent effect of YA on ABTS radical scavenging activity. * *p* < 0.05 compared to control (0 μg/mL). (**B**) Decrease in blood EtOH concentration by YA pre-administration. (**C**) ADH activity increased by YA pre-administration. (**D**) ALDH activity increased by YA pre-administration. (**E**) Catalase activity in liver tissue increased by YA pre-administration. (**F**) Decrease in CYP2E1 mRNA expression and CYP2E1 activity in liver tissue in YA combined group. Blood and liver tissue were obtained from mice at 5 h of EtOH administration. Each bar represents mean ± SD of five independent experiments. Figures B through E share bar graph labels. ^#^ *p* < 0.05 compared to vehicle; * *p* < 0.05 compared to EtOH + saline group; ^†^ *p* < 0.05 compared to EtOH + OH.

The effect of YA dipeptide, a main component of OH, was investigated in a single high dose of EtOH administered to mice. YA showed a dose-dependent increase in ABTS radical scavenging activity (n = 5, *p* < 0.05, Figure 7A). YA significantly decreased EtOH concentrations (n = 10, *p* < 0.05, Figure 7B) and increased ADH (n = 10, *p* < 0.05, Figure 7C) and ALDH (n = 10, *p* < 0.05, Figure 7D) activities compared to the EtOH + saline group (*p* < 0.05). In liver tissue obtained from EtOH-intoxicated mice, YA pre-administration significantly increased catalase activity (n = 5, *p* < 0.05, Figure 7E), and decreased CYP2E1 expression level and activity compared to the EtOH-only administered group (EtOH + saline) (n = 5, *p* < 0.05, Figure 7F). However, the effect of YA was lower than that of OH on EtOH metabolism, indicating that other components contained in OH work together to enhance EtOH metabolism. The OH effect was more than 20% higher than the YA effect on EtOH metabolism. 

## 3. Discussion

Research on the effectiveness of a single high dose of alcohol is relatively small. Numerous studies have shown that chronic exposure to EtOH damages the liver. This study was designed to identify the effects of a single high dose of alcohol on the liver and the protective effect of OH and YA against alcohol intoxication by screening for alcohol-metabolizing enzymes. Interestingly, we found that a single high dose of EtOH induced oxidative and inflammatory stress in the liver, and that OH and YA reduced the oxidative and inflammatory stress. 

A hangover, with unpleasant physical and mental symptoms, occurs when there is a bout of heavy alcohol intake, usually when it exceeds 1.0 g EtOH/kg BW. We produced a single high dose of EtOH administered to a rodent model by oral administration of 3.0 g EtOH/kg BW. The EtOH-intoxicated rodents showed increased blood EtOH concentration and poor motor performance compared to the vehicle group administered saline. Motor performance was impaired in proportion to EtOH concentration, which was still high up to 5 h after administration. Impaired motor performance means incomplete recovery from EtOH intoxication [25]. Pre-administration of OH reduced blood EtOH concentration and restored motor performance faster compared to the EtOH + saline group. A high concentration of OH (200 mg/mL) is the most effective in reducing EtOH concentration compared to low and medium concentrations (50 mg/mL and 100 mg/mL) in vivo. These results indicate that OH may be effective in enhancing alcohol metabolism. More rapid and efficient metabolism of EtOH may be associated with less severe liver toxicity. 

The OH pre-administered group (EtOH + OH) showed high activity in alcohol-metabolizing enzymes such as ADH, ALDH, and catalase in blood compared to the EtOH + saline group. Thus, blood acetaldehyde and EtOH concentrations were markedly decreased in the EtOH + OH group. Higher ADH and catalase and ALDH activity can quickly lower EtOH and acetealdehyde concentration, respectively. ADH and ALDH mainly metabolize EtOH in the liver through the oxidative pathways. CYP2E1 and catalase also break down EtOH to acetaldehyde, but they are inducible with high dose of alcohol and chronic alcohol intake [26]. Most studies have demonstrated that acetaldehyde, a highly toxic substance, induces oxidative stress in excessive chronic alcohol consumption by activating NADPH oxidase [9], damages mitochondria, and leads to degeneration of hepatocytes [27]. CYP2E1 is a main pathway for regulating EtOH-induced oxidative stress. ROS generated by CYP2E1 rapidly enter and damage cell membranes, and contribute to the pro-inflammatory profile of alcohol-induced liver damage [27,28,29]. In our EtOH-intoxicated rodents, CYP2E1 was upregulated in mRNA expression and activity. CYP2E1 mRNA expression and CYP2E1 activity were decreased in the EtOH + OH group. However, some studies have shown that EtOH increases liver CYP2E1 activity and protein expression without increasing CYP2E1 mRNA levels [30]. On the other hand, similar to our results, in vitro and in vivo studies demonstrate that both acute and chronic EtOH administration increases CYP2E1 mRNA and protein expression, resulting in liver toxicity [31,32,33]. The increase in CYP2E1 mRNA expression and activity is most likely a response to oxidative stress after an acute high dose of EtOH administration. OH pre-administration reduced oxidative stress in liver tissue. Catalase, located in peroxisomes, is known to play a much smaller role in alcohol oxidation than ADH or CYP2E1, but high dose of alcohol or chronic alcohol intake may increase its role. Pre-administration of OH markedly increased catalase activity compared to the EtOH + saline group. In EtOH-intoxicated rats, the magnitude of the increase in ADH and ALDH activity by OH was less than the difference in EtOH clearance rates. These results suggest that OH may affect catalase and CYP2E1 as well as ADH and ALDH activity. Moreover, OH reduced EtOH-induced ROS and inflammatory cytokines. It is believed that all of the OH functions mentioned here contribute to the EtOH clearance.

The levels of AST and ALT in serum are the most useful tools for diagnosing liver damage. Liver damage is primarily associated with increased ALT present in the liver. AST is present not only in the liver, but also in skeletal and cardiac muscles [34]. When the cytoplasm and mitochondria of hepatocytes are severely damaged, AST is released into the blood stream, increasing blood levels compared to ALT [35]. OH pre-administration showed no significant difference among experimental groups, vehicle and EtOH + saline. However, apoptotic signals were detected in liver tissue obtained from EtOH-intoxicated rats. Alcohol metabolism via ADH, ALDH, CYP2E1, and catalase increases ROS production, causing mitochondrial damage [36]. Increased ROS activates the mitochondrial apoptosis pathway, including increased Bax/Bcl2 ratio, cytochrome c release, caspase-9, -3, -6, and -7 activation, and PARP cleavage. In addition, a high dose of EtOH increases the expression of toll-like receptor 4 and releases large amounts of ROS and inflammatory mediators that can accelerate inflammatory and oxidative damage [37]. In the EtOH + saline group, changes in the level of AST in the blood were not observed, but tissue damage was observed. This seems to indicate that the results obtained in one measurement time did not sufficiently reflect the changes in blood and liver tissue. We measured blood ALT and AST concentration at 1, 3, and 5 h after EtOH administration. Usually, 6 h has been used for evaluation of ALT and AST levels [34,38]. In mice injected intraperitoneally with a single EtOH dose, ALT and AST were increased at 8 h and 12 h after administration, respectively [39]. Compared to other studies focusing on liver damage, we focused on measuring the activity of EtOH metabolic enzymes, so the blood analysis time was slightly faster. Therefore, the test may not have been able to confirm the effect of OH on the concentration of ALT and AST in the blood.

We used 2% (*v*/*v*) EtOH, which induced approximately 50% cell death in Chang liver cells. The concentration of EtOH used in this study is higher than that used to induce cell death in other studies. In general, 100 mM is used to induce cell injury. However, even in reports, 100 mM EtOH induces cell death approximately 25% of the time [40,41]. These results are similar to our results. Although the method of displaying concentration is different, 100 ~ 200 mM EtOH induces ~30% cell death. In line with our study, cell death is induced by ~50% in human normal liver cells L-02 treated with 400 mM EtOH for 24 h [41]. The 2% EtOH used in this study is a very high concentration with 50% cytotoxicity. OH reduced the strong cytotoxicity of EtOH in a concentration-dependent manner. This effect of OH may result in reducing apoptotic signals in the livers obtained from EtOH-intoxicated rats.

**Figure 8:**
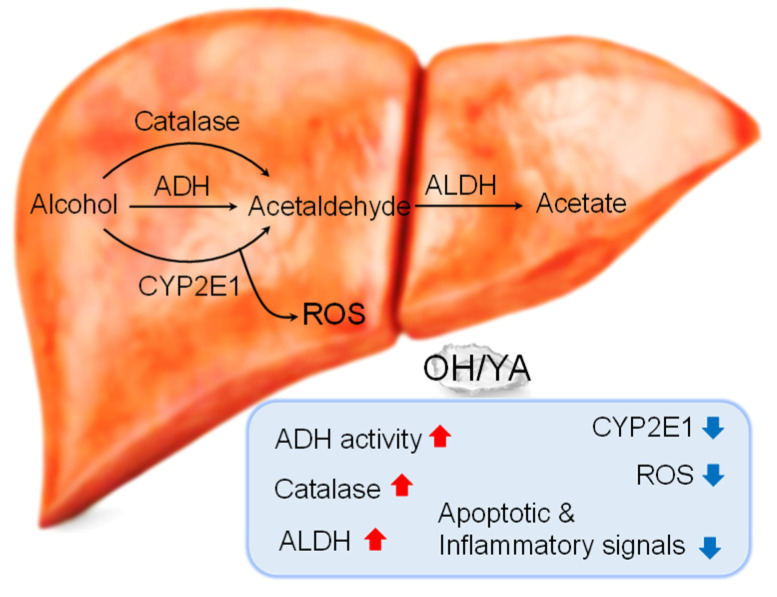
Schematic representation of OH/YA effect on liver of a single high dose of EtOH administered to rodents. OH and YA increased the activity of alcohol metabolic enzymes, such as ADH, catalase, and ALDH, which are involved in relieving hangovers. In addition, OH and YA reduced EtOH-induced increase in CYP2E1 mRNA expression and activity, ROS generation, and apoptotic and inflammatory signals.

OH enhanced alcohol metabolism through increased ADH, ALDH, and catalase activity and decreased CYP2E1 expression/activity, ROS/inflammatory mediators, and apoptotic signals in EtOH-intoxicated rodents (Figure 8). OH is a product extracted from oysters by an enzymatic hydrolysis process. Oysters have long been eaten by people in many ways, including raw, grilled, smoked, fried, steamed, canned, and boiled, and are used in a variety of drinks. Another product of oysters is hydrolysate, where bioactive peptides are released from the parent protein by enzymatic hydrolysis [42]. Thus, OH has higher functional and physiological activity than oyster or its extract [16]. The antioxidant and anti-inflammatory effects of OH are likely to be exhibited by the YA dipeptide, which is a main component released from oyster by an enzymatic hydrolysis process. However, the YA effect on EtOH metabolism was smaller than OH effect, suggesting that other components in OH may accelerate EtOH metabolism. OH is a complex of several peptides, so it will work better than a single peptide YA. Phenylalanine-tyrosine-asparagine (FYN), threonine-alanine-tyrosine (TAY), lysine-tyrosine (KY), and valine-lysine (VK) are also identified in OH [43]. These peptides can contribute to OH effects, such as anti-inflammatory, antioxidant, antihypertensive, and anti-alcohol effects. 

Many bioactive compounds with antioxidant and anti-inflammatory activity are known to be effective in alleviating hangovers and liver damage, because oxidative stress and inflammation are implicated as key mediators of hangover symptoms and liver damage [9,44]. Consistent excessive EtOH intake and accumulated hangover symptoms are considered to be the main causes of liver disease and have a negative impact on the economy. Thus, it is necessary to develop functional foods that quickly relieve symptoms related to hangovers, minimize side effects, and are easily accessible in the diet. Nonsteroidal anti-inflammatory drugs have been prescribed to alleviate hangover symptoms [45]. In addition, the HDD used to compare the hangover-relieving effects of OH is commercialized in Korea, and many people take it before and after drinking alcohol. However, there are side effects of hovenia extracts. In particular, if there is liver damage, the weakened liver function may worsen the damage. There was a case of toxic hepatitis by hovenia [46]. In this study, the effect of OH was better than HDD on reducing liver toxicity. In addition, it is suggested that the EtOH-metabolizing effect of oysters could be manifested by the peptides contained in OH. The amount of peptides produced when eating oysters is less than when OH or peptide is consumed directly, but oysters have the advantage that they can be eaten easily in a meal.

At this point, we do not know the exact mechanism by which oyster hydrolysate and YA promote alcohol metabolism. However, based on the results in which amino acids and minerals regulate ADH and ALDH activity [20,47], we suggest that various amino acids and minerals rich in OH [12,13] are likely to contribute to promoting EtOH metabolism. YA, the main peptide of OH, is also expected to contribute to enhancing EtOH metabolism. OH and YA will contribute to liver protection by enhancing EtOH metabolism through regulation of EtOH-metabolizing factors such as ADH, ALDH, catalase, and CYP2E1. These are also involved in the reduction of EtOH-induced oxidative and inflammatory stress. Further study is needed to determine how OH and YA regulate alcohol metabolism enzymes.

There are some limitations in this study. In our previous study, five angiotensin I-converting enzyme inhibitory peptides, including YA, were purified and identified from OH [43]. However, in this study, the activity of YA in alcohol metabolism and hepatoprotection could not be compared with other peptides isolated from OH. It is necessary to compare the effects of peptides isolated from OH in further studies. It is also necessary to test whether OH/peptides affect ADH and ALDH activity in vitro. In addition, this study focused on the OH effect on EtOH metabolism and hepatoprotection, based on previous studies showing that the hydrolysates containing a high concentration of peptide have a higher physiological effect compared to the parent protein. It was necessary to first test the effect of oyster extract or lyophilized oyster powder untreated with hydrolase to determine whether it has similar activity as OH. Although many reports have demonstrated the hepatoprotective effect of oyster extract, the method of making oyster extract differs between reports, and some comments have been omitted [48,49,50]. So, we were unable to accurately determine the effect of oyster extract. 

Animal model studies are disadvantageous compared to human hangover studies in that they cannot determine the association between severity of the subjective hangover and specific behavioral and/or biochemical indicators [7]. However, animal models have the advantage of being able to identify various pathological changes in hangover processes. Although the results of this study are somewhat far from human hangovers, it is believed that they may contribute to hangover relief by enhancing alcohol metabolism. The definition of alcohol hangover has recently been updated to “the combination of negative mental and physical symptoms which can be experienced after a single episode of alcohol consumption, starting when blood alcohol concentration approaches zero” [1]. The content of this study seems to be somewhat different from the definition of alcohol hangovers because it is defined as drinking more than one’s own dose, not binge drinking. However, binge drinking also causes hangovers, so our findings could provide information on binge drinking.

## 4. Materials and Methods 

### 4.1. Preparation of Oyster Hydrolysate and YA Peptide

Crassostrea gigas (length, 5.8 ± 0.4 cm; height, 3.2 ± 0.4 cm; body weight (BW), 9.8 ± 2.1 g) was collected in 2018–2019 at a fish farm in Tongyeong (Korea), frozen, and stored for 1-2 years. The preparation of the oyster hydrolysate was done according to a previous protocol with slight modification [43]. Frozen oysters were thawed in tap water, rinsed, and boiled in water at 100 °C for 30 min to remove the smell and salt. They were ground with a meat mill (M-12S, Hankook Fujee Industry, Hwaseong, Korea) equipped with a 5-mm-diameter hole plate, and suspended twice in tap water. Transglutaminase (Ajinomoto Co., Tokyo, Japan) was added to form a protein bridge and reacted at 30 °C with stirring. Protamex (Biosis, Busan, Korea) was added at 1% (w/w) of the crushing weight and hydrolyzed at 40 °C for 1 h and further hydrolyzed with 1% Neutrase (Biosis, Busan, Korea) at 50 °C for 1 h with stirring. The mixture was heated at 100 °C for 30 min to inactivate the enzyme, and filtered through a 200 mesh filter. The filtrate was concentrated at 100 °C until about 30 Brix using a concentrator (HS-1000 L, Hansung F&C Co., Incheon, Korea) and then dried by a spray dryer (20K, Yoojin Tech Co., Pyeongtaek, Korea) in a co-current air flow mode. It was stored at −70 °C and used for the experiment.

Since the active ingredient of the oyster hydrolysate is set to peptide, the amino acid sequence of the purified peptide fragment was determined using LC/MS/MS. Amino acid sequenced peptides are synthesized with a purity of 95% or higher to test their function. In addition, methods to quantify functional and effective peptides have been developed and validated. In protein hydrolysates, it is important to determine how much peptide per gram contains as an indicator/active ingredient. Synthetic YA product was purchased from Sigma-Aldrich (St. Louis, MI, USA).

### 4.2. Cell Culture

Chang liver cells and RAW264.7 macrophage were obtained from American Type Culture Collection (ATCC, Manassas, VA, USA). Cells were cultured in Dulbecco’s Modified Eagle’s Medium (DMEM; Gibco/Life Technologies, Grand Island, NY, USA) supplemented with 10% fetal bovine serum (FBS; Gibco/Life Technologies), 100 U/mL penicillin (Gibco), and 100 mg/mL streptomycin (Gibco). The cells were incubated at 37 °C in 95% air and 5% CO_2_ gas mixture. The medium was replaced every 2 days.

### 4.3. Cell Viability Assay

Cell viability of Chang liver and RAW264.7 cells was determined using a 3-(4,5-dimethylthiazole-2-yl)-2,5-diphenyl tetrazolium bromide (MTT) reagent (5 mg/mL in phosphate buffered saline (PBS), Duchefa Biochemie, Haarlem, The Netherlands). As previously mentioned, the MTT assay procedure was performed [51].

### 4.4. Measurement of DPPH and ABTS Radical Scavenging Activity

The 2.2-diphenyl-1-picrylhydrazyl (DPPH) and the 2,2-azinobis-(3-ethylbenzothiazoline-6-sulfonate) (ABTS) radical-scavenging activities of OH were evaluated according to the previous protocols [17].

### 4.5. Measurement of Cyclooxygenase-2 (COX-2) and 5-Lipoxygenase (5-LO) Inhibition Activity

The percentages of COX-2 inhibition and 5-LO inhibition were measured according to previous protocols [17].

### 4.6. Live/Dead Cell Staining 

Chang liver cells at a density 3 × 10^3^ /well were seeded in a poly-L-lysine-coated glass-bottomed culture dish (SPL, Pocheon, Korea) and incubated at 37 °C and 5% CO_2_ for 48 h. Then, the cells were treated with 2% EtOH and 100 µg/mL OH for 24 h. As previously mentioned, the staining procedure was performed [51].

### 4.7. Measurement of Intracellular Reactive Oxygen Species (ROS) Levels in Cells

Intracellular ROS generation was determined using dichlorodihydrofluorescein (H_2_DCFDA, Calbiochem, San Diego, CA, USA). Chang liver cells were plated in a poly-L-lysine-coated confocol image dish and incubated with 5 μM H_2_DCFDA at 37 °C for 30 min in the dark. As previously mentioned, the measurement of ROS levels was performed [52]. 

### 4.8. Measurement of Mitochondrial Membrane Potentials (MMP)

MMP changes were measured by a JC-1 mitochondrial membrane potential detection kit (Biotium Inc. Hayward, CA, USA). Chang liver cells at a density of 3 × 10^3^ /well were seeded in a poly-L-lysine-coated confocol image dish for recoding MMP. As previously mentioned, the measurement of MMP was performed [51]. 

### 4.9. Measurement of Intracellular Ca^2+^ Concentration

Intracellular Ca^2+^ concentration ([Ca^2+^]_i_) was measured using the IX70 Fluoview confocal laser scanning microscope equipped with a fluorescence system (Olympus). Chang liver cells cultured on the poly-L-lysine-coated confocol image dish were incubated with 5 μM Fluo-3AM (Thermo Fisher Scientific, Rockford, IL, USA) in serum-free DMEM media for 30 min and washed three times with PBS. As previously mentioned, the measurement of [Ca^2+^]_i_ was performed [51].

### 4.10. Alcohol Intoxication Model

Animal experiments were performed in accordance with the guidelines of the Gyeongsang National University animal care and use committee (GNU-190308-R0013 and GNU-200702-M0041). Male Sprague Dawley rats (8 weeks old) and C57BL/6 mice (8 weeks old) were purchased from Koatech Co. (Animal Breeding Center, Pyongtaek, Korea). Animals were maintained under a 12 h light/dark cycle in a specific pathogen-free area with food and water freely available in the animal facility for 1 week prior to the experiment. All experimental animals were randomly separated into four groups as follows: vehicle (saline + saline), EtOH + saline (3 g/kg), EtOH + OH (200 mg/kg), and EtOH + hovenia dulcis drink (HDD, hovenia 16 mg/mL) for rats and EtOH + tyrosine-alanine (YA, 50 mg/kg) for mice. EtOH was administered, and saline, OH, HDD, and YA were pre-administered 1 h prior to EtOH administration by oral gavage. Blood was collected from the tail vein at 0, 1, and 3 h after EtOH administration and from the heart at 5 h after EtOH administration. Liver tissues were quickly isolated and placed into a deep freezer at −80 °C or a 4% paraformaldehyde solution for further experimentation.

### 4.11. Rotarod Performance Test

Motor coordination and balance were evaluated in a rotarod apparatus (B1001-006, B.S Technolab Inc., Seoul, Korea). The rotarod apparatus consists of five lanes with 60 mm rotor diameter, 75 mm lane width, 240 mm falling height, and 290 mm lane separator diameter. All rats were evaluated on the rotarod with the rotation set at 10 to 15 revolutions per minute (rpm). To test their performance, rats were placed on the rotating cylinder at a 45° angel with an initial rotation speed of 10 rpm. If the rat falls, the rotating cylinder timing is stopped. The falling time was recorded for each lane and trial.

### 4.12. Measurement of Ethanol (EtOH) Concentration

Measurement of serum EtOH concentration was performed using an Ethanol Assay Kit (Abcam) according to the manufacturer’s protocol. Samples were prepared by 1:10 dilution with ethanol assay buffer. Standard curve was prepared using 1 mM ethanol standard and making serial dilution of 0, 2, 4, 6, 8, and 10 nmol/well. Reaction mix at 50 μL per well was prepared by mixing 46 μL assay buffer, 2 μL ethanol probe, and 2 μL ethanol enzyme cocktail. Reaction mix was added into standard and sample wells and incubated at 37 °C for 30 min in the dark. The absorbance was measured at 570 nm using the VERSAmax™ microplate reader (Molecular Devices). Ethanol concentration (mM) was calculated by dividing the ethanol concentration obtained from the standard curve by the sample volume used in the well, and was adjusted to sample dilution factor. Serum EtOH concentration was also measured by GC Labs (Yongin, Korea). EtOH concentration was analyzed by an enzymatic assay and expressed as a percentage.

### 4.13. Detection of Acetaldehyde Concentration

The concentration of acetaldehyde was measured using an EnzyChrom^TM^ Acetaldehyde Assay Kit according to the manufacturer’s protocol (BioAssay Systems, Hayward, CA, USA). Acetaldehyde standard was prepared in 0, 0.6, 1.2, and 2 mM per well by diluting 3 M acetaldehyde standard. Standard and serum samples (20 µL) were added into a 96-well plate, and 80 µL of working reagent (assay buffer, NAD/MTT, and enzymes A and B) was added into each well containing standard and sample, then incubated for 30 min at room temperature. The absorbance was measured at 565 nm using the VERSAmax™ microplate reader (Molecular Devices). Acetaldehyde concentration (mM) was calculated by subtracting the absorbance of the blank from sample absorbance. Final concentration was adjusted to sample dilution factor.

### 4.14. Measurement of Alcohol Dehydrogenase (ADH) Activity 

ADH activity in serum was measured using an Alcohol Dehydrogenase Assay Kit (Abcam) according to the manufacturer’s protocol. Serum samples were prepared by 1:10 dilution with ADH assay buffer, and reaction mix was prepared by mixing 82 µL ADH assay buffer, 8 µL developer, and 10 µL isopropanol. Standard curve was prepared in 0, 2, 4, 6, 8, and 10 nmol per well by diluting 1 mM NADH standard. Reaction mix (50 µL) was added into standard and sample wells, then incubated for 3 min at room temperature. The absorbance was measured every 5 min for 120 min at 450 nm. ADH activity (mU/mL) was calculated by dividing the amount of NADH generated by ADH by the product of multiplying the reaction time and the sample volume used in the well. Final concentration was adjusted to the sample dilution factor.

### 4.15. Measurement of NAD-Dependent Aldehyde Dehydrogenase (ALDH) Activity 

ALDH activity in serum was analyzed by an ALDH Activity Assay Kit (Abcam) according to the manufacturer’s protocol. Briefly, samples were prepared by 1:10 dilution with ALDH assay buffer, and reaction mix was prepared by mixing 43 μL ALDH assay buffer, 2 μL ALDH substrate mix, and 5 μL acetaldehyde. Standard curve was prepared in 0, 2, 4, 6, 8, and 10 nmol per well by diluting 1 mM NADH standard. Reaction mix (50 μL) was added into standard and sample wells, then incubated for 5 min at room temperature. The absorbance was measured every 5 min for 60 min at 450 nm. ALDH activity (mU/mL) was calculated by dividing the amount of NADH generated by samples by the product of multiplying the reaction time and the sample volume used in the well. Final concentration was adjusted to the sample dilution factor.

### 4.16. Measurement of Catalase Activity 

Liver tissues was homogenized in 50 mM phosphate buffer (pH 7.4) containing 1% Triton X-100 and the homogenates were subjected to centrifugation at 12,000× *g* for 20 min (Eppendorf Centrifuge 5424R, Eppendorf AG, Hamburg, Germany). After centrifugation, the supernatant was transferred to a clean tube and provided as tissue lysates. Catalase activity was measured as previously described [17] with slight modification. Briefly, 1.99 mL of 50 mM phosphate buffer (pH 7.4), 1 mL of 30 mM H_2_O_2_, and 10 µL of tissue lysates were mixed and incubated at room temperature for 5 min. The absorbance was measured immediately at 240 nm using a UV spectrophotometer (Biochrom Ltd., Cambridge, UK) and provided as OD-1. The absorbance of the reaction was measured again after 5 min and provided as OD-2. Catalase activity was calculated by subtracting the value of OD-2 from OD-1. A 50 mM phosphate buffer (pH 7.4) was used as a blank prior to measurement.

### 4.17. Measurement of CYP2E1 Activity

Liver tissues (500 mg) homogenized in 5 mL of 0.15 M KCl were sonicated for 10 s in an ice bath with a VCX-500 Ultrasonic Processor (Sonics and Materials Inc., Newton, CT, USA). Tissue lysate was subjected to centrifugation at 9000× *g* for 15 min, and the supernatant was further centrifuged at 105,000× *g* for 1 h using the Optima XL-100K Ultracentrifuge (Beckman, Palo Alto, CA, USA). The microsomal pellet was collected and resuspended in 200 µL of 0.15 M KCl. Protein concentration in microsomal lysates was quantified using a Pierce^TM^ bicinchoninic acid (BCA) protein assay kit (Thermo Fisher Scientific). Microsomal protein (100 µg) was incubated in 100 µL reaction buffer (0.1 M KH_2_PO_4_/K_2_HPO_4_, pH 7.4, 0.4 mM p-nitrophenol, 1 mM NADPH) at 37 °C in a shaking water bath for 1 h. At the end of incubation, 30 µL of 20% trichloroacetic acid (TCA) was added to stop the reaction, and the mixture was subjected to centrifugation at 10,000× *g* for 10 min. The supernatant was collected and mixed with 10 µL of 10 N NaOH. The absorbance of the supernatant was measured at 510 nm using the VERSAmax™ microplate reader (Molecular Devices). CYP2E1 activity (pmol/mg/min) was calculated by multiplying sample absorbance by an extinction coefficient of 9.53 × 10^5^ M^−1^·cm^−1^.

### 4.18. Measurement of Alanine Aminotransferase (ALT) and Aspartate Aminotransferase (AST) Levels

ALT and AST levels in serum were measured by GC Labs (Yongin, Korea), which uses the International Federation of Clinical Chemistry standard method. ALT and AST levels were analyzed using the measured enzymatic colorimetric method with no added pyridoxal phosphate. In a test tube, 1 mL of substrate solution (AST, 2 mmol/L α-ketoglutarate and 200 mmol/L aspartate; ALT, 2 mmol/L α-ketoglutarate and 200 mmol/L alanine) and 0.2 mL of serum were added and incubated at 37 °C for 30 min (ALT) or 1 h (AST). Immediately after the completion of the reaction, 1 mL of a coloring solution (2,4-dinitrophenyl hydrazine, 1 mmol/L) was added, followed by incubation at room temperature for 20 min, and then 10 mL of 0.4 N NaOH was added to measure the absorbance at 520 nm (Modular Analytics, Roche, Germany). Distilled water was used as a blank.

### 4.19. Measurement of Total Free Radical Activity in Tissues

Total free radical activity was measured using the Oxiselect^TM^ In Vitro ROS/RNS assay kit (Cell Biolabs, San Diego, CA, USA) according to the manufacture’s protocol. Briefly, DCF standards were prepared in a concentration range of 0–10 μM by diluting the 1 mM DCF stock in 1 × PBS. Then, 50 µL of tissue lysates or DCF standard was added into a 96-well black-bottomed fluorescence plate (Nunclon^TM^, Thermo Fisher Scientific, Roskilde, Denmark), and 50 µL of 1 × catalyst was added into the wells. Then the wells were mixed and incubated for 5 min at room temperature. DCFH solution (100 µL) was added to the wells and incubated at room temperature for 45 min in the dark. The fluorescence was measured using a GloMax^®^ Explorer (Promega, Madison, WI, USA) at 480 nm excitation and 530 nm emission. This assay employs a proprietary quenched fluorogenic probe, dichlorodihydrofluorescin DiOxyQ (DCFH-DiOxyQ), which is a specific ROS/RNS probe. The probe reacts with ROS/RNS species, which are rapidly oxidized to fluorescent DCF. Fluorescence intensity is proportional to the total ROS/RNS levels within the sample.

### 4.20. Detection of Oxidative Stress in Tissue 

Oxidative stress in liver tissue was analyzed an OxyIHC^TM^ oxidative stress detection kit (Millipore, Burlington, MA, USA) according to the manufacturer’s protocol. The tissue slides were deparaffinized, rehydrated, and then incubated with 250 μL of 1 × Antigen Retrieval Buffer for 20 min at 37 °C. The tissues were then allowed to cool down for 15 min at room temperature, then washed three times with Wash Buffer for 5 min. DNPH solution was added to the tissue sections and the tissue slides were incubated in a humidified chamber for 30 min at room temperature in the dark. The tissue sections were washed with 1 × Wash Buffer three times and then incubated with 1 × Blocking Buffer in a humidified chamber for 30 min at room temperature. Primary antibody was added to the tissue section for overnight at 4 °C. After primary antibody incubation, sections were washed with 1 × Wash Buffer three times for each for 5 min. The sections were then incubated with biotinylated secondary antibody in a humidified chamber for 30 min at room temperature, then washed three times. To quench endogenous peroxidases, tissue sections were treated with 3 % H_2_O_2_ for 10 min at room temperature. The slides were washed three times with 1 × Wash Buffer for 5 min. The tissue sections were exposed to streptavidin conjugated horseradish peroxidase (HRP) and incubated for 30 min at room temperature, washed three times, exposed to DAB/A-B mixture for 3 min, and washed three times. For counterstaining, tissue slides were treated with hematoxylin for 3 min. The sections were then processed for dehydration through a graded series of alcohols (70% to 100% ethanol, 3 min each), cleared in xylene, and mounted with Permount Mounting Medium (Fisher Chemical, Geel, Belgium). The sections were observed and photographed using a BX61VS microscope (Olympus, Tokyo, Japan).

### 4.21. RT-PCR and Real-Time PCR

Total RNA was isolated from Chang liver cells and rodent liver tissues and was used to synthesize first-strand cDNA using a reverse transcriptase kit (DiaStartTM RT kit; SolGent, Daejeon, Korea) for RT-PCR and real-time PCR. As previously mentioned, the procedures for RT-PCR and real-time PCR were performed [51]. 

### 4.22. Western Blot Analysis

Total protein was isolated from liver tissue using the RIPA buffer (25 mM Tris-HCl (pH 7.4), 150 mM NaCl, 1% NP-40, 1% deoxycholate, 0.1% sodium dodecyl sulfate (SDS); Thermo Fisher Scientific) containing 1 × protease inhibitor cocktail (Roche Diagnostics, Indianapolis, IN, USA). The tissue lysates were incubated on ice with intermittent vortexing for 30 min and were clarified at 16,600× *g* (13,000 rpm; Eppendorf AG) at 4 °C for 20 min by centrifugation. The supernatant was isolated and stored at −70 °C until use. Mitochondrial and cytosolic fractions were isolated using a mitochondria isolation kit for tissue (Thermo Fisher Scientific) according to the manufacturer’s protocol. Liver tissue was homogenized in 800 µL of ice-cold PBS using T-18 Basic Ultra Turrax Homogenizer (IKA Inc., Wilmington, NC, USA), and the homogenized tissue was centrifuged at 1000× *g* for 3 min at 4 °C using the Eppendorf Centrifuge 5424R (Eppendorf AG). The pellets were suspended with 800 µL of bovine serum albumin (BSA)/Reagent A solution, vortexed for 5 sec, and incubated on ice for 2 min. Reagent B (10 µL) was added to the suspension, vortexed for 5 sec, and incubated for 5 min on ice. Reagent C (800 µL) was added to the suspension and then inverted several times. The suspensions were centrifuged at 700× *g* for 10 min at 4 °C. The supernatants were transferred to new tubes and subjected to centrifugation at 3000× *g* for 15 min at 4 °C. The resulting supernatants (cytosolic fractions) were stored at −80 °C for further analysis. The pellets were washed twice with 500 µL of Wash Buffer and subjected to centrifugation at 12,000× *g* for 5 min. The pellets were resuspended in 200 µL of 2% 3-[(3-cholamidopropyl)dimethylaminonio]-1-propanesulfonate hydrate (CHAPS) in Tris buffered saline (TBS; 25 mM Tris, 0.15 M NaCl, pH 7.2) and were vortexed for 1 min, followed by centrifugation at 13,000× *g* for 2 min. The resulting supernatants (mitochondrial fractions) were stored at −80 °C for further analysis.

Measurements of protein concentration in tissue lysates and electrophoresis procedure were performed as previously described [53]. The membranes blocked with 5% (*w*/*v*) fat-free dry milk in TBS with tween-20 at room temperature for 60 min were incubated with anti-Bax (1:200 dilution; Santa Cruz Biotechnology, Dallas, TX, USA), anti-Bcl-2 (1:200 dilution; Santa Cruz Biotechnology), anti-cytochrome C (1:1000; Cell Signaling, Danvers, MA, USA), anti-VDAC (1:1000; Cell Signaling), anti-caspase-3 (1:1000; Cell Signaling), and anti-β-actin antibody (1:5000 dilution; Thermo Fisher Scientific) at 4 °C overnight. After the primary antibody incubation, they were incubated with a secondary HRP-conjugated anti-rabbit or anti-mouse antibody at 1:10,000 (Assay Designs, Ann Arbor, MI, USA). Immuno-positive bands were enhanced with chemiluminescence (EzWestLumi plus; ATTO Gentaur, Tokyo, Japan) and visualized using the iBright^TM^ CL1500 imaging system (Thermo Scientific Fisher/ Life Technologies Holdings Pte Ltd., Singapore). 

### 4.23. Measurement of Caspase 3/7 Activity

Liver tissues were homogenized in ice-cold RIPA buffer (25 mM Tris-HCl; pH7.4, 150 mM NaCl, 1% NP-40, 1% sodium deoxycholate, 0.1% SDS) containing DTT at 10 mM final concentration in the absence of protease inhibitors that could interfere with the assay. As previously described, measurement of caspase 3/7 activity was performed [53]. 

### 4.24. Measurement of Leukotrienes Level

Leukotriene D4 (LTD4) and E4 (LTE4) levels were measured using ELISA kits according to the manufacturer’s protocol (MyBioSource, San Diego, CA, USA). As previously described, measurement of leukotrienes level was performed [54].

### 4.25. Data Analysis and Statistics

The images of the Western blots and agarose gel were captured using an iBright^TM^ CL1500 imaging system (Thermo Scientific Fisher/ Life Technologies Holdings Pte Ltd.). The bands were quantified by ImageJ software (version 1.49, National Institutes of Health, Bethesda, MD, USA). Data are presented as mean ± standard deviation (SD). One-way ANOVA/Bonferroni test or the Kruskal–Wallis/Mann–Whitney test were selected after the normality test to analyze differences among groups (OriginPro2020, OriginLab Corp., Northampton, MA, USA). In addition, one-way repeated measures ANOVA/Tukey test was applied for multiple tests performed over a period of time on the same group (Figure 4A and 4B, OriginPro2020). A *p* < 0.05 was considered as the statistical significance criterion. 

## 5. Conclusions

Our findings spark new awareness regarding a single high dose of EtOH intake. Excessive alcohol intake seems to cause liver toxicity regardless of frequency. Pre-administration of OH helps to reduce liver damage by accelerating alcohol metabolism and removing ROS. YA, a main component of OH, is partially responsible for the OH effect on EtOH metabolism. Our findings suggest that OH and YA may be potential functional foods and ingredients that can contribute to reduction of EtOH toxicity in liver by enhancing alcohol metabolism.

## Figures and Tables

**Table 1:** Reduced alcohol concentration in alcohol-intoxicated rats by high concentrations of OH.

Group	Alcohol Concentration and Liver Enzymes at 5 h after EtOH Injection		
	Alcohol (%)	ALT (U/L)	AST (U/L)
Vehicle	0.00 ± 0.00	57.44 ± 13.80	186.11 ± 47.06
EtOH + saline	0.10 ± 0.04 ^a^	101.86 ± 37.42 ^a^	297.29 ± 120.73
EtOH + LOH	0.08 ± 0.04 ^a^	91.67 ± 31.69	227.86 ± 54.35
EtOH + MOH	0.05 ± 0.03	88.25 ± 38.08	216.83 ± 38.27
EtOH + HOH	0.03 ± 0.02 ^b^	89.53 ± 33.18	269.15 ± 84.59

^a^*p* < 0.05 compared to vehicle group. ^b^ *p* < 0.05 compared to EtOH + saline group. LOH, MOH, and HOH: low (50 mg/kg), medium (100 mg/kg), and high (200 mg/kg) concentrations of OH. ALT, alanine aminotransferase; AST, aspartate aminotransferase.

**Table 2:** No changes in liver-to-body weight, ALT, and AST levels by OH pre-administration.

Group	BW (g)	LW/BW (g)	LW/BW	ALT(U/L)	AST(U/L)
Vehicle	202.5 ± 3.54	6.97 ± 0.07	0.03 ± 0.00	59.75 ±12.67	191.50 ± 41.34
EtOH + saline	203.7 ± 1.15	7.19 ± 0.06	0.04 ± 0.00	94.90 ± 32.29 ^a^	272.55 ± 99.76
EtOH + OH	202.7 ± 2.08	6.87 ± 0.15	0.03 ± 0.00	80.17 ± 24.87	248.78 ± 80.18
EtOH+ HDD	205.3 ± 3.51	6.59 ± 0.44	0.03 ± 0.00	64.60 ± 6.50 ^b^	181.43 ± 34.23

BW, body weight; LW, liver weight. ^a^ *p* < 0.05 compared to vehicle group; ^b^ *p* < 0.05 compared to EtOH + saline group.

**Table 3:** Primer sequences used for PCR.

Gene Name	GenBank	Primer Sequences (5′-3′)	Expected Size (bp)	Application
	Accession No.			
*CYP2E1*	NM_031543.1	Sense: GGTGGAGGAGCTCAAAAAGAC	313	RT-PCR
		Antisense: GTCCAGTGACTGAAGGTGTTC		
*IL-1β*	NM_031512.2	Sense: TGGCAGCTACCTATGTCTTGC	152	Real-time PCR
		Antisense: CAGTGCAGCTGTCTAATGGGA		
		Sense: GAAAACGTGTGTTTCCCTCC	265	RT-PCR
		Antisense: GATGTGCT GTGCTTCATTC		
*IL-6*	NM_012589.2	Sense: AAGAGACTTCCAGCCAGTTGC	115	Real-time PCR
		Antisense: TGGTCTGTTGTGGGTGGTATC		
*TNF-α*	NM_012675.3	Sense: CAGGAGAAAGTCAGCCTCCTC	100	Real-time PCR
		Antisense: CCAGGTACATGGGCTCATACC		
		Sense: GGCTGTACCTTATCTACTCCC	300	RT-PCR
		Antisense: TGACTCCAAAGTAGACCTGC		
*GAPDH*	NM_017008	Sense: CATGGCCTTCCGTGTTC	103	Real-time PCR
		Antisense: CTGCTTCACCACCTTCTT		
		Sense: CTAAAGGGCATCCTGGGC	201	RT-PCR
		Antisense: TTACTCCTTGGAGGCCATG		

## Abbreviations

EtOH	Ethanol
OH	Oyster hydrolysate
YA	Tyrosine-Alanine
